# Retraction Note to: miR-665 promotes hepatocellular carcinoma cell migration, invasion, and proliferation by decreasing Hippo signaling through targeting PTPRB

**DOI:** 10.1038/s41419-023-05654-x

**Published:** 2023-02-09

**Authors:** Yuanchang Hu, Chao Yang, Shikun Yang, Feng Cheng, Jianhua Rao, Xuehao Wang

**Affiliations:** Hepatobiliary/Liver Transplantation Center, The First Affiliated Hospital of Nanjing Medical University, Key Laboratory of Living Donor Transplantation, Chinese Academy of Medical Sciences, Nanjing, Jiangsu Province China

Retraction Note to: *Cell Death and Disease* 9: 954 (2018) 10.1038/s41419-018-0978-y, published online 20 September 2018

The Editors have retracted this article. After publication, concerns were raised regarding overlap in the cell and tissue images presented in the figures, as well as the cell lines used in the study. Specifically:

- There appears to be overlap in Fig. 2A and B between images representing different groups.
- In Fig. 2C, the NC and pre-miR-665 0 h images appear to originate from the same sample.
- There appears to be overlap in Fig. 5C and D between images representing different groups.
- In Fig. 8E and F, the PTPRB images in the pre-miR-665 group appear to contain an overlapping area.
- L02 and SMMC-7721 cell lines have been reported to be contaminated with HeLa and are therefore unsuitable for liver cancer research.
- HepG2 cells have been reported to be unsuitable as a model of hepatocellular carcinoma.

Additionally, there is no patient consent statement in the article.

The authors have stated that the image errors occurred due to incorrect data storage.

The Editors therefore no longer have confidence in the presented data and the ethics of the study.

Yuanchang Hu has stated on behalf of all co-authors that they agree to this retraction.

